# Frailty in Patients With Hematologic Malignancies and Patients Undergoing Hematopoietic Stem Cell Transplantation: A Systematic Review

**DOI:** 10.1002/cnr2.70456

**Published:** 2026-01-09

**Authors:** Marit Bakken, Marie Roko Kallager, Marie Hamilton Larsen, Simen A. Steindal, Kristin J. Skaarud

**Affiliations:** ^1^ Department of Haematology Oslo University Hospital Oslo Norway; ^2^ Department for Postgraduate Studies Lovisenberg Diaconal University College Oslo Norway; ^3^ Institute of Nursing VID Specialized University Oslo Norway

**Keywords:** frailty, geriatric assessment, hematologic malignancies, hematopoietic stem cell transplantation, systematic review

## Abstract

**Background:**

Hematopoietic stem cell transplantation (HSCT) is associated with significant morbidity and mortality. Frailty further increases these risks in recipients of HSCT. This systematic review analyzes the extent of frailty in patients with hematologic malignancies and patients undergoing HSCT, and explores the associations between frailty and age, and clinical outcomes.

**Methods:**

CINAHL (EBSCO), Embase (Ovid), and Medline were searched for quantitative studies including assessment tools aimed at identifying frailty or vulnerability. Two reviewers independently assessed eligibility, extracted data from the included articles, performed a quality appraisal, and analyzed the findings through narrative synthesis.

**Results:**

Of the 5190 abstracts screened, 17 articles involving 17 different tools describing frailty were identified. Frailty was characterized as abnormal nutritional status, comorbidities, and an impact on social support, physical activity, and mental health. Frailty was associated with increased age but was also shown in younger patients. Moreover, frailty was associated with worse clinical outcomes.

**Conclusions:**

Patients with hematologic malignancies and patients undergoing HSCT were at risk of frailty, and frailty was associated with older age and worse clinical outcomes.

## 1 Introduction

The aging population of patients with hematologic malignancies, eligible for hematopoietic stem cell transplantation (HSCT), has a high risk of frailty [1, 2]. Frailty is characterized by a decline in age‐related multiple physiological functioning, resulting in increased vulnerability and reduced ability to withstand acute stressors, such as hematological malignancies, chemotherapy, and HSCT [2, 3]. The conditioning regimen administered before stem cell infusion varies on a continuum from high‐dose (myeloablative) to reduced‐intensity to non‐myeloablative. The higher intensity of the conditioning regimen is often associated with increased toxicity [4]. The introduction of reduced‐intensity conditioning has led to a rise in HSCT, specifically in older patients and patients with comorbidity [5]. The major adverse outcomes of HSCT are relapse of the underlying disease, toxicity of the conditioning regimen, infections, and graft‐versus‐host disease (GvHD) [6]. Over the past three decades, treatment‐related mortality has been reduced; however, survival decreases with age [7, 8].

Screening patients with hematological malignancies for frailty pre‐HSCT may greatly enhance health personnel's ability to identify those at risk of frailty, allowing for timely intervention before treatment begins and reducing the risk of adverse outcomes [2]. Traditionally, decisions regarding patient selection and the optimal time point to perform HSCT depend on the risk stratification of the underlying disease, chronological age, and comorbidities. Tools such as the hematopoietic cell transplant comorbidity index (HCT‐CI) [9] and the European Group for Blood and Marrow Transplantation risk score (EBMT‐score) are commonly used to aid in such a decision‐making process [10]. However, chronological age, comorbidity, and performance status may have limited utility in capturing the heterogeneity of older patients with hematological malignancies. In addition, comorbidity indices, such as the HSCT‐CI and EBMT scores, may not adequately identify frailty or physical and psychological vulnerability and impairments, factors that could significantly impact morbidity and mortality after HSCT [1]. Thus, there is a growing need to supplement standard risk assessment with measures of physical and psychological functioning [1]. Multiple assessment tools are used to identify frailty, impairments, or vulnerabilities in patients with hematological malignancies and those undergoing HSCT [2]. Frailty is multidimensional, including physical and psychosocial factors. Individuals may fluctuate between states of severity of frailty, such as vulnerable, unfit, prefit, or frail [1]. Frailty can be evaluated by generic geriatric assessment (GA) tools, such as the clinical frailty scale (CFS), the vulnerable elders survey‐13 vulnerability score (VES‐13), or by more disease‐specific tools, such as the revised myeloma comorbidity index (R‐MCI), the International Myeloma Working Group frailty score (IMWG frailty score), or a combination of these [1]. This additional layer of assessment can aid in determining which patients may benefit from HSCT and who is most likely to have an adverse outcome [2].

Three systematic reviews reported the results of GA in patients with hematological malignancies and in patients undergoing HSCT [11, 12, 13]. Typically, GA tools contain domains such as cognition, physical function, comorbidities, polypharmacy, social support, mental health, and nutritional status. Based on GA scores, patients were classified into groups of the extent of frailty or vulnerability (e.g., fit, prefrail/unfit/intermediate‐fit, or frail) [12]. One systematic review, including patients with hematological malignancies, found that several geriatric impairments and frailty were predictive of shorter overall survival rates. This review suggests that GA assessment, even in patients with a good performance status, may detect impaired geriatric domains, which may be predictive of mortality. Moreover, these geriatric impairments propose a higher risk of treatment‐related toxicity, treatment non‐completion, and increased utilization of healthcare services [13]. This is in line with a systematic review of patients with hematological malignancies. Geriatric impairments are associated with shorter overall survival, poor physical activities, nutritional status, and cognitive capacities [11]. Comorbidity, physical capacity, and nutritional status retained their significance in multivariate analyses, whereas age and performance status lost their predictive value in most studies [11]. A systematic review and meta‐analysis of patients with myeloma found that the risks of hematologic adverse events were similar in intermediate‐fit and frail patients. However, a significantly increased risk of non‐hematologic adverse events was found in frail patients compared to fit patients. Patients classified as frail showed a higher risk of death than fit patients [12]. A narrative review of GA and the management of cancer patients in general emphasized that GA may not cover unspecific complications such as delirium, falls, and functional decline, and that older patients are a heterogenous group [14]. This suggests that older adults undergoing HSCT might be fitter and younger than the traditional older adult population. Moreover, intensive chemotherapy before HSCT in middle‐aged patients has been associated with frailty. Previous systematic reviews assessing frailty screening in patients with hematological malignancies and in patients undergoing HSCT have mainly focused on GA tools [7, 14, 15, 16]. Thus, there is a need to include multiple tools when assessing frailty and clinical outcomes, and the interplay between frailty and age in these patients [14, 17]. This broader approach can provide a more comprehensive understanding of frailty in patients with hematological malignancies and patients undergoing HSCT. This systematic review aimed to analyze the extent of frailty in patients with hematologic malignancies and those undergoing HSCT, and to explore the associations between frailty and age, and clinical outcomes.

## 2 Methods

This systematic review was reported according to the synthesis without meta‐analysis (SWiM) guidelines [18] (Supporting Information). A systematic search was conducted using the databases CINAHL (EBSCO), Embase (Ovid), and Medline from inception until January 31, 2024. The search strategy was built in Medline by the first authors based on keywords and subject headings used in previous publications, and on the advice from a research librarian. The search strategy consisted of two elements: (1) hematological malignancy and stem cell transplantation and (2) frailty and vulnerability. The search strategy was then piloted in medline, and the search strategy was applied to the other databases (Supporting Information). Moreover, we hand‐searched the reference lists of the included studies to ensure that we did not miss any relevant studies. The inclusion and exclusion criteria are listed in Table 1.

**TABLE 1:** Inclusion and exclusion criteria.

	Inclusion	Exclusion
Type of studies	Quantitative studies (cross‐sectional, randomized controlled trials and cohort studies) published in peer review journals	Case—control studies, qualitative studies, all types of reviews, comments, letters, conference abstracts, not full‐text articles
Population	Patients > 18 years with a hematologic malignancy	Non‐hematologic malignancies
Phenomena of interest	Frailty in patients with hematologic malignancies and patients undergoing HSCT identified using assessment tools aimed at identifying frailty or vulnerability	Assessment tools used to identify other conditions than frailty
Period	Inception to January 31, 2024	After January 31, 2024
Language	English	Not published in English

First, the results of our search were transferred to Endnote20, and duplicates were eliminated. Second, the results were transferred to Rayyan [19], a web‐based collaboration software platform that helps organize and blind the screening. In Rayyan, an automatic duplicate check identified more duplicates, which were subsequently removed.

The first authors (M.B. and M.R.K.) independently screened the title and abstract for eligibility. The same two authors then independently assessed the full‐text publications. Any disagreements between these authors (M.B. and M.R.K.) were resolved through discussion until an agreement was reached. The first authors (M.B. and M.R.K.) independently used Joanna Briggs Institute (JBI) critical appraisal tools to evaluate the methodological quality of the included studies. Cohort studies were assessed using the JBI checklist for cohort studies, and cross‐sectional studies were assessed using the JBI checklist for analytical cross‐sectional studies [20]. Disagreements were resolved by discussion until agreement was ascertained. No articles were excluded based on the quality appraisal (Supporting Information). Data from the included articles were independently extracted by the first authors (M.B. or M.R.K.) using multiple standardized data collection forms. Data included author, year, country, study methods, patients' characteristics, description of the assessment tools, and main findings related to the aim of this review. Subsequently, the data were ascertained for accuracy against the original papers. No disagreements in data extraction occurred during this process. We used narrative synthesis to analyze and summarize what is known about the study aim [21]. The narrative synthesis was guided by the European Social Research Council Guidance on the Conduct of Narrative Synthesis in systematic reviews [22]. We analyzed and summarized data from multiple studies by organizing data according to standardized data collection forms and used text and words to summarize and explain the findings of the included studies [22, 23]. We compared the data from the data collection forms to identify similarities and differences across the included articles.

## 3 Results

**FIGURE 1:**
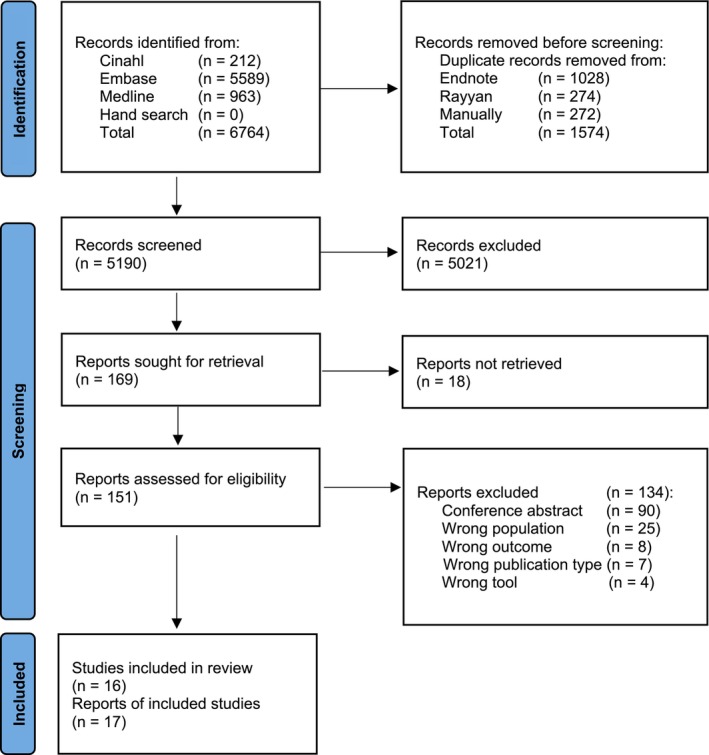
Preferred reporting items for systematic reviews and meta‐analyses (PRISMA) flow diagram shows articles of frailty in HSCT/hematologic malignancies identified in our systematic search.

The initial search yielded 6764 publications. After the removal of duplicates and the screening of titles and abstracts (*n* = 5190), 151 full‐text publications were reviewed. One hundred and thirty‐four studies were excluded. The reasons for exclusion are described in Figure 1. No relevant studies were found by hand search. Seventeen articles based on 16 studies were included in this review.

### 3.1 Article Characteristics

The articles were published between 2013 and 2024 and were conducted in the United States (*n* = 11), Germany (*n* = 2), Italy (*n* = 2), Brazil (*n* = 1), and Canada (*n* = 1). Fifteen articles were cohort studies [24, 25, 26, 27, 28, 29, 30, 31, 32, 33, 34, 35, 36, 37, 38] and two were cross‐sectional studies [39, 40]. Patients were recruited from single‐center or multicenter sites, and the number of participants varied between 50 and 801. The characteristics of the included articles are presented in Table 2.

**TABLE 2:** Literature matrix.

Author, (year), country (reference number)	Population	Aim	Design	Study limitations	Outcome/results frailty
Aydin et al. (2023) Italy [24]	*n* = 197, age ≥ 60 with AML.	To investigate the capacity of fitness by using Sorror and G8 score and determine overall survival before combining them with AML‐score for complete remission.	Retrospective cohort study.	Used two different protocols for induction therapy.	Significant impact on overall survival in AML‐score, G8 score and HCT‐CI score. G8: Median overall survival for fit patients was 20.7 months, compared to 6.7 months for unfit patients. HCT‐CI: Median overall survival among fit patients was 16.1 months, compared to 6.7 months for unfit patients. The significance remained for AML‐score and G8 score (not for HCT‐CI score) in multivariable analysis.
Belotti et al. (2020) Italy [25]	*n* = 131, age ≥ 65, newly diagnosed symptomatic MM.	To investigate the impact of age, and the predictive value of IMWG frailty score, Charlton comorbidity index, and HSCT‐CI score for progression free survival.	Prospective cohort study.	Small sample size. GA does not include gait speed. No analyses of the population receiving double HSCT and those receiving reduced dose of melphalan.	Frail patients were not considered for HSCT. Frail patients had significantly worse outcomes compared to fit and unfit patients. No significant differences between fit and unfit patient regarding outcome. Fit and unfit patients undergoing HSCT had better progression free survival compared to fit and unfit patients not undergoing HSCT. Patients aged 65–69 fared better than those aged ≥ 70. International staging system III and ADL ≤ 4 and/or iADL ≤ 5 (not age) independently affected progression free survival in multivariable analysis. IMWG frailty score seems to be more effective and objective, compared to clinical decision making.
Derman et al. (2019) The US [26]	*n* = 247, age ≥ 43, with ALL, AML, MDS, MPN, NHL and other diagnoses.	GA‐guided multidisciplinary team established a Transplant Optimization Program and report on the intervention to investigate, evaluate and enhance resilience of older adult candidates for HSCT and cellular therapy.	Retrospective observational cohort study.	Single institutional study of heterogeneous patients. Limited subset analysis.	Vulnerabilities found by GA did not differ between auto‐ and allo‐HSCT patients, even though the patients undergoing auto‐HSCT had a higher median age. Pre‐transplant optimization program group presented significant higher proportions with IADL impairments (49.2% vs. 29.5%) and frail 4 m walk (31.7% vs. 5.9%) compared with the transplant optimization program multidisciplinary team clinic.
Engelhardt et al. (2017) Germany [27]	*n* = 801, age ≥ 21 with MM	To develop and validate the MM‐specific risk score R‐MCI, and to investigate differences in overall survival and progression free survival.	Prospective cohort study.	Data for the R‐MCI group was generated from a single center. Patients got different treatments.	Frailty was associated with severe comorbidity in 62% of the patients. Frailty was presented in the younger cohort and increases with age. 13.5% were frail according to R‐MCI versus 25.1% according to initial myeloma comorbidity index. 56% were intermediate‐fit with R‐MCI, compared to 41% with the initial myelomacomorbidity index. The fit group (*n* = 247), median overall survival of 10.1 years in the training set, vs. not reached in the validation set. In the intermediate‐fit group (*n* = 446), overall survival was 4.4 years in the training set, versus 5.9 years in the validation set. In the frail group (*n* = 108), overall survival was 1.2 years in the training set versus 0.8 years in the validation set. Multivariate analysis determined renal, lung and Karnofsky performance status impairment, frailty and age as significant risks for overall survival.
Holler et al. (2023) Germany [28]	*n* = 250, age ≥ 27, with MM or AL‐amyloidosis received induction treatment.	To investigate if using an objective risk score in adaptation of therapy intensity, rather than by a physician alone, could improve therapy efficacy and avoid therapy toxicities.	Prospective cohort study.	Do not specify if the results are from the entire cohort, or specific for the transplanted. Single center. Heterogenous age categorization.	R‐MCI may predict serious adverse events. Patients aged ≥ 70 years, and patients aged 60–69 years had similar overall survival, progression free survival and serious adverse events, compared to patients aged ≤ 60 years. Increased median age in the intermediate‐fit and frail patients. Patients classified as fit and intermediate fit had significant better overall survival and progression free survival compared to frail patients. Serious hematological adverse events per patient increased from 0.23 in fit, 0.72 in intermediate‐fit and 1.1 in frail patients compared to 0.23, 0.48 and 1.13 for non‐hematological serious adverse events. R‐MCI may assist in anticipating the likelihood of serious adverse events. No difference in leukocytopenia in frail and intermediate‐fit patients, while anemia and thrombocytopenia were observed in frail patients. Significant increases in different subgroups with infectious, renal, pulmonary, and cardiac serious adverse events in frail patients, compared with fit and intermediate‐fit patients.
Holmes et al. (2014) The US [39]	*n* = 134, age ≥ 60, with AML/MDS, CLL, other diagnoses considered for allo‐HSCT.	To investigate if the VES‐13 and the G8 screening tool score may identify those who are likely to have an abnormal GA or the presence of frailty syndrome.	Cross‐sectional study.	Limited sample size. Single institution study.	33 (66%) patients had an abnormal GA, 11 (22%) were frail at baseline. Domains were abnormal nutrition, comorbidity, low physical activity, low grip strength, slow gait speed, exhaustion and social support. 42 (84%) had at least one impairment. Five had abnormal VES‐13, 28 had abnormal G8. Both correlated with GA. The G8, but not VES‐13, correlated with the frailty index. G8 had a higher sensitivity for frailty, while VES‐13 had a higher specificity.
Huang et al. (2023) The US [29]	*n* = 148, age ≥ 50, with AML, MDS, ALL, NHL, MPF/MPNs, planned to undergo allo‐HSCT.	To investigate if cGA and a global frailty index could predict the risk of cognitive decline after allo‐HSCT in older patients.	Longitudinal prospective cohort study.	Small sample size. Low rates of frailty. Some patients had already cognitive impairments measured at baseline.	Patients classified as frail at baseline were 7.4 times more likely to develop cognitive decline after 1 year. Other domains were elevated BMI, limitations in walking and ADL. Overall survival after 1 year: 71% in the non‐frail group, 59% in the pre‐frail group and 56% in the frail group. BOMC‐score was significantly associated with frailty at 6 months. The pre‐frail and frail patients had worse BOMC‐score compared to non‐frail.
Lew et al. (2022) The US [30]	*n* = 180, age ≥ 18, with leukemia, lymphoma, MDS/MPN, other diagnoses considered for HSCT.	To investigate if GA could identify functional impairments before HSCT.	Prospective cohort study.	The follow‐up was limited. Small sample size since patients with impairments did not receive HSCT. The patients were healthier and fitter.	Of the 157 evaluated with C‐POP, 127 (80.9%) had at least one impairment, 50 (31.8%) had two impairments, 28 (17.8%) had three impairments, 4 (2.5%) had four impairments. Physical impairment dominated. Patients aged 18–39 were more likely to have a physical impairment, while patients aged ≥ 60 were more likely to have a cognitive impairment. Nutritional impairment occurred in 47 (29.9%) patients and was most common in patients aged ≥ 60 (31.4%), followed by 18–39 (30.8%) and 40–59 (27.9%). GA before HSCT identified candidates for HSCT among older patients. Post‐HSCT, 53.6% had improved physical impairment, 50% improved cognitive function, and 61.9% improved nutritional status. 30 patients (40.5%) had a bloodstream infection post‐HSCT. Post‐HSCT patients with nutritional impairment and older age had significantly worse overall survival.
Muffly et al. (2013) The US [31]	*n* = 228, age ≥ 50 and older, with AML, MDS, NHL, ALL, CML, CLL, other diagnoses scheduled to undergo allo‐HSCT.	To investigate if CGA could describe the severity and prevalence of vulnerabilities in patients eligible for HSCT.	Prospective pilot cohort study.	Single center pilot study. Different condition protocols and donor sources. 50 years and older resulted in a younger cohort.	CGA uncovered impairments in frailty among older patients undergoing HSCT. Increased frailty was significantly associated with worse performance status. 28% of the patients had an impairment of walking speed, 22% had an impairment of grip strength, 31% experienced exhaustion, 60% had weight loss and 31% had a decline in physical activity. No significant association between older age and increased limitation by standard HSCT measures or CGA. No difference in low IADL disability, physical function, low mental function, frailty and undernutrition was found in patients aged 50–59 compared with ≥ 60.
Muffly et al. (2014) The US [32]	*n* = 203, age ≥ 50, with AML, MDS, NHL, ALL, CML, CLL, other diagnoses planned to undergo allo‐HSCT.	To report the significance of the use of GA before HSCT in a large cohort of older HSCT patients.	Prospective cohort study.	≥ 50 years resulted in a younger cohort than usually used in a typical GA study. Heterogeneity, such as age, diagnosis, condition regimes. Whether the impairments before HSCT were due to the diagnosis or the induction treatment was unknown.	Older age was significantly associated with increased comorbidity. Older age, limited IADL, poor mental health and slow walk speed were significantly associated with decreased overall survival. GA has a predictive prognostic value before HSCT. Domains were grip strength, physical exhaustion, walk speed, mental health and systemic inflammation.
Nathwani et al. (2020) The US [33]	*n* = 165, age ≥ 65, newly diagnosed with MM or relapsed, at four different sites.	To investigate a tablet‐based mGA for evaluation of feasibility, usability and acceptability, and evaluate the physician use of and perceived utility of the mGA to determine treatment decision. The secondary aim was to explore the relationship between treatment outcomes and frailty.	Observational prospective pilot cohort study.	The results may underestimate the frequency of toxicity due to lack of prospective follow up in a therapeutic clinical trial.	Significant associations between patients classified as fit, and patients considered as eligible for HSCT, and between patients classified as frail with patients considered as ineligible for HSCT. No significant association between toxicity and frailty status.
Ombres et al. (2022) The US [34]	*n* = 134, age ≥ 60, with AML/MDS, CLL, other diagnoses undergoing allo‐HSCT.	To investigate the prevalence of frailty in older patients at baseline and post‐HSCT and investigate if the baseline assessment was associated with frailty or death at 3 and 6 months after allo‐HSCT.	Prospective cohort study.	Small sample size. Single center study. Pilot study.	Older age was associated with abnormal GA. At baseline, 11 (22%) patients were frail, while at 3 months, 22 (55%) were either frail or deceased. Of these, 14 (63.6%) were not frail at baseline. Fit patients at baseline may develop frailty during treatment. Neither frailty nor abnormal GA at baseline were associated with frailty or death at follow‐up.
Pamukcuoglu et al. (2019) The US [35]	*n* = 140, age ≥ 40, with acute leukemia, lymphoma, MM were due to undergo HSCT.	To investigate if frailty at baseline assessed by Fried criteria was associated with mortality, and grades 3 to 4 non‐ hematologic toxicities in HSCT recipients 1‐year after HSCT, and whether age is associated with the outcomes.	Prospective longitudinal cohort study.	Heterogeneous patients (i.e., both auto and allo‐HSCT). Some of the selected indices were based on self‐report that is, differed from Fried criteria.	Frailty was not associated with age. Patients classified as frail were more likely to experience toxicity (86% vs. 70% non‐frail), organ‐specific toxicity and a higher risk to develop non‐neutropenic infections and pneumonia, disorders in the nervous system and overall mortality compared with non‐frail patients. No significant differences between type of transplant (auto vs. allo‐HSCT) or the intensity of the conditioning (MAC vs. RIC) for the severity of toxicity. Better overall survival in non‐frail patients (81%) compared with frail patients (48%) 1‐year post‐HSCT.
Rodrigues et al. (2020) Brazil [40]	*n* = 40, age ≥ 60, with AML, MDS, lymphoma, MPF, mycosis planned for HSCT.	To investigate the results of a cGA before allo‐HSCT.	Cross‐sectional, retrospective analysis.	Small sample size. Single center. Retrospective analysis.	cGA could predict vulnerabilities before HSCT. Domains were impaired physical activity, impaired cognitive function, and abnormal nutrition. *p*‐values not reported.
Salas et al. (2021) Canada [36]	*n* = 280, age ≥ 19, with AML, MDS, MPN, ALL, lymphoproliferative disease, CMML, AA (*n* = 3), chronic neutrophilic leukemia planned to undergo allo‐HSCT.	To investigate a newly designed assessment of frailty and functionality, who was implemented for allo‐HSCT, and describe characteristics, feasibility, and efficacy of this assessment.	Prospective cohort pilot study.	Small sample size. Limited follow‐up.	Frailty was not associated with overall survival, but with worse non‐relapse mortality. Worse overall survival and non‐relapsed mortality were associated with timed up and go test‐score > 10 s and raised CRP. Patients age ≥ 50 with abnormal grip strength and elevated CRP had significantly higher risk for worse outcome. Timed up and go test‐score > 10 s and raised CRP remained significant for worse overall survival and non‐relapse mortality in multivariable analysis. Poor score on self‐rated health was a predictor of non‐relapse mortality.
Smith et al. (2022) The US [37]	*n* = 110, age ≥ 19, with leukemia, MDS, other diagnoses planned to undergo HSCT.	To investigate the association between frailty, domain‐ specific neurocognitive performance and clinical outcomes in HSCT patients.	Prospective cohort study.	Small sample size. Limited follow‐up regarding longitudinal data. Short follow up after HSCT.	Higher levels of frailty were associated with poorer cognitive function and lower executive function. Frailty was associated with longer length of stay, not associated with overall survival. No association between age and frailty, nor an association between age and poorer cognitive function.
Sung et al. (2024) The US [38]	*n* = 280, age ≥ 60, with acute leukemia, MDS, MPN planned to undergo HSCT at three different sites.	To investigate if FFP before HSCT is associated with overall survival after HSCT in older patients.	Prospective cohort study.	Small sample size.	The median age for the fit group was 66, for the pre‐frail group 67 years, and for the frail group 68 years. Age impacts frailty. Overall survival was significantly worse for patients classified as frail and pre‐frail compared to patients classified as fit. They also had higher rates of sepsis, acute graft‐versus‐host disease, and pneumonia. FFP predicted overall survival after HSCT in older patients. Patients aged ≥ 70 compared to patients aged 60–69 had higher risk of non‐relapse mortality (37.5% vs. 20%), bacteremia/sepsis (11.3% vs. 2.5%), aGvHD (10% vs. 6%) and pneumonia (3.8% vs. 1.5%).

Abbreviations: ADL, activity of daily living (needing help with simple activities); aGvHD, acute graft‐versus‐host disease; ALL, acute lymphocytic leukemia; AML, acute myelogenous leukemia; AML score, Acute Myelocytic Leukemia score; BMI, body mass index; BOMC‐score, blessed orientation‐memory‐concentration score; cGA, comprehensive geriatric assessment; CCA, cancer‐specific geriatric assessment; C‐POP, clinical pre transplantation optimization program; CRP, C‐reactive protein; FFP, fried frailty phenotype; G8 score, geriatric‐8 score; GA, geriatric assessment; HCT‐CI, hematopoietic cell transplantation‐specific comorbidity index; IADL, instrumental activities of daily living (needing help with more complex skills correlating with ability to live alone); IMWG frailty score, International Myeloma Working Group frailty score; MDS, myelodysplastic syndrome; mGA, modified cancer specific geriatric assessment; MoCA, montreal cognitive assessment; MM, multiple myeloma; MPN, myeloproliferative neoplasms; MPF, myelofibrosis; NHL, non‐Hodgkin lymphoma; R‐MCI, revised myeloma comorbidity index; SPPB, short physical performance battery; VES‐13, vulnerable elders survey.

#### 3.1.1 Participant Characteristics

The results of the quality assessment are provided in Supporting Information. The participants were hospitalized for a variety of hematologic malignancies. Patients with acute myelogenous leukemia and myelodysplastic syndrome were represented in most articles (*n* = 12) [24, 26, 29, 30, 31, 32, 34, 36, 37, 38, 39, 40]. Fore articles reported cases of leukemia but did not specify the leukemia subtype [30, 35, 37, 38]. Seven articles included patients with lymphoma (*n* = 7) [26, 29, 30, 31, 32, 35, 40] and multiple myeloma (*n* = 5) [25, 27, 28, 33, 35].

Most of the articles (*n* = 15) described patients' eligibility for HSCT, while two articles did not clearly describe whether patients were eligible for HSCT [27, 38]. Two articles reported that all included patients underwent HSCT [29, 37]. The majority of the articles included patients treated with allo‐HSCT (*n* = 11) [24, 26, 29, 30, 31, 32, 34, 36, 37, 39, 40], while four articles included patients undergoing auto‐HSCT [25, 27, 28, 33]. Derman et al. [26] included patients who underwent either allo‐HSCT or auto‐HSCT, while in the remaining two articles, the number of patients treated with HSCT was not clear [35, 38].

The ages of the participants ranged from 18 to 93 years, and eight articles presented their results based on the categorization of different age groups [26, 30, 31, 32, 36, 37, 38, 40]. The median age varied between 54.7 and 72.3 years. Four studies did not report median age [30, 31, 34, 38]. One article presented results based on age groups and the prevalence of frailty [25]. The patient characteristics are described in Table 3.

**TABLE 3:** Patient characteristics in the included studies.

Author, (year), reference number	Patient population (age range)	Median age	Diagnosis	*n*	Patients eligible for HSCT *n* (%)^a^	Recipients of HSCT *n* (%)^a^
Aydin et al. (2023) [24]	60–77	68	AML, MDS, myeloid sarcoma	197	120 (61%)	Auto: 0 Allo: 33 (27%)
Belotti et al. (2020) [25]	65–75	70.7	MM	131	85 (65%)	Auto: 72 (85%) Allo: 0
Derman et al. (2019) [26]	43–83 < 50, *n* = 2 50–59, *n* = 14 60–69, *n* = 138 ≥ 70, *n* = 93	67.9	ALL, AML, MDS, MPN, NHL	247	240 (92%)	Auto: 31 (27%) Allo: 85 (73%)
Engelhardt et al. (2017) [27]	21–93	63	MM	801	Unclear	Auto: 383 Allo: unclear
Holler et al. (2023) [28]	27–92	62	MM, AL‐amyloidosis	250	179 (72%)	Auto: 179 Allo: 0
Holmes et al. (2014) [39]	60–73	65.4	AML, MDS, CLL, other	134	50	Auto: 0 Allo: 48
Huang et al. (2023) [29]	50–76	62	AML, MDS, ALL, NHL, MF, other	148	148	Auto: 0 Allo: 148
Lew et al. (2022) [30]	18–60+ (not specified) 18–39, *n* = 35 40–59, *n* = 68 > 60, *n* = 77	Not reported	MDS/MPN, Leukemia, Lymphoma, other	180	157	Auto: 0 Allo: 77 (49%)
Muffly et al. (2013) [31]	50–70+ not specified 50–59, *n* = 100 60–69, *n* = 62 70+, *n* = 4	Not reported	AML, MDS, NHL, CML, CLL, other	228	166	Auto: 0 Allo: 166
Muffly et al. (2014) [32]	50–73	58	AML, MDS, NHL, ALL, CML, CLL, other	271	203	Auto: 0 Allo: 203
Nathwani et al. (2020) [33]	65–95	72.3	MM	165	52	Auto: 52 Allo: 0
Ombres et al. (2022) [34]	60–70+ (not specified)	Not reported	AML/MDS, CLL, other	50	48	Auto: 0 Allo: 48
Pamukcuoglu et al. (2019) [35]	40–73	59	Acute leukemia, lymphoma, multiple myeloma, other	140	117	Auto: unclear Allo: unclear
Rodrigues et al. (2020) [40]	60–76 60–64, *n* = 14 65–70, *n* = 13 > 70, *n* = 13	67.6	AML, MDS, lymphoma, myelofibrosis, mycosis fungoides	52	40	Auto: 0 Allo: 40
Salas et al. (2021) [36]	19–77 19–50, *n* = 59 51–64, *n* = 66 > 65, *n* = 43	58	AML, MDS, MPN, ALL, lymphoproliferative disease, CMML, CML, AA, CNL	280	168	Auto: 0 Allo: 168
Smith et al. (2022) [37]	19–75 < 50 50–60 60+, *n* = not specified	54.7	Leukemia, MDS, other	110	110	Auto: 0 Allo: 64
Sung et al. (2024) [38]	60–78 60–69, *n* = 200 70–78, *n* = 80	Not reported	Acute leukemia, MDS, MPN	280	Unclear	Auto: 0 Allo: unclear

Abbreviations: AA, aplastic anemia; AML, acute myeloid leukemia; AL‐amyloidosis, amyloidosis light chain; ALL, acute lymphocytic leukemia; Allo, allogeneic; Auto, autologous; CLL, chronic lymphocytic leukemia; CML, chronic myelogenous leukemia; CMML, chronic myelomonocytic leukemia; CNL, chronic neutrophilic leukemia; HSCT, hematopoietic stem cell transplantation; MDS, myelodysplastic syndrome; MF, myelofibrosis, MPN, myeloproliferative neoplasms; NHL, non‐Hodkin lymphoma.

^a^ Percentages were reported when provided by the articles.

#### 3.1.2 Frailty Assessment Tools and Their Domains

Across the articles, 17 different tools were used to describe frailty, vulnerability, or impairments. Eleven tools identified frailty: acute myelocytic leukemia score (AML‐score), CFS, comprehensive geriatric assessment (CGA), deficit accumulation frailty index (DAFI), fried frailty index (FFI), Geriatric‐8 score (G8‐score), GA, hematopoietic cell transplantation‐specific comorbidity index (HCT‐CI), IMWG frailty score, R‐MCI, and short physical performance battery (SPPB). Five tools identified impairments: blessed orientation‐memory‐concentration test (BOMC‐test), cancer‐specific GA (cGA), clinical pretransplantation optimization program (C‐POP), modified cancer‐specific GA (mGA), and montreal cognitive assessment (MoCA), and one tool identified vulnerability: VES‐13. The tool used by most studies was the FFI (*n* = 8), followed by the IMWG frailty score (*n* = 3). One article reported a significant association between worse MoCA score and worse SPPB score in multivariable analyses [37].

Abnormal nutrition status, comorbidities, impact on social support, cognitive impairments, and activities of daily living, as well as low physical activity, low grip strength, low gait speed, weight loss, and exhaustion were described as characteristics of frailty in 10 articles [26, 29, 30, 31, 32, 34, 36, 37, 39, 40]. The two domains of low grip strength [26, 27, 31, 32, 34, 35, 38, 39, 40] and weight loss [24, 27, 31, 32, 34, 35, 38, 39, 40] were each reported in nine articles. The domains of physical activity, exhaustion, and low gait speed were reported in eight articles [27, 31, 32, 34, 35, 38, 39, 40]. Seven articles reported comorbidities [25, 26, 28, 29, 33, 36, 40], five articles reported abnormal nutrition status [26, 29, 30, 35, 40], and five articles reported activities of daily living [25, 28, 29, 33, 40] as domains of frailty. In four articles, cognitive impairments were reported [29, 30, 37, 40], while three articles reported the domain impact on social support [26, 29, 35]. The characteristics of each tool are described in Table 4.

**TABLE 4:** Description of the assessment tools used in the included studies.

Tool	Description	References
AML‐score	Acute myelocytic leukemia score. Domains: clinical and laboratory variables (age, body temperature, hemoglobin, platelets, lactate dehydrogenase, fibrinogen, de novo vs. secondary leukemia), variables of cytogenetic and molecular risk	Aydin et al. (2023) [24]
BOMC‐test	Blessed orientation‐memory‐concentration test. Domains: orientation, memory, concentration/attention	Huang et al. (2023) [29]
cGA	Cancer‐specific geriatric assessment. Domains: functional status, comorbidities, cognition, nutrition, mental health and social support	Huang et al. (2023) [29]
CGA	Comprehensive geriatric assessment. Domains: activities of daily living, instrumental activities of daily living, hand‐grip test, timed up and go test, performance status (Karnofsky performance status and eastern cooperative oncology group performance status), falls, comorbidity hematopoietic cell transplantation comorbidity index, polypharmacy, frailty (fried phenotype criteria), cognition and depression (mini‐mental status exam; loss of memory, verbal fluency, clock test, geriatric depression scale), nutritional status (mini nutritional assessment) and body mass index.	Rodrigues et al. (2020) [40]
CFS	Clinical frailty score. Domains: primary hematological disorder, comorbidities, function and associated features	Salas et al. (2021) [36]
C‐POP	Clinical pre‐transplantation optimization program. Domains: physical function (falls questionnaire, SPPB, 30‐s sit‐to‐stand, 6‐min walk test, fried frailty index), cognitive assessment (MoCA), Nutrition assessment (perioperative nutrition screening) and psychological assessment (PTSD screening, patient health questionnaire).	Lew et al. (2022) [22]
DAFI	Deficit accumulation frailty index. Domains: marital status, instrumental activities of daily living, medical outcomes study, physical functioning, Karnofsky performance status, time up and go test, number of falls, mental health inventory, nutrition, comorbidities other cancer, arthritis, glaucoma, emphysema, hypertension, heart disease, circulation, diabetes, gastrointestinal, osteoporosis, liver/kidney, stroke, depression, eyesight, hearing, number of medications, social support.	Huang et al. (2023) [29]
Fried frailty index (FI)/fried criteria/frailty score (fried)/fried phenotype criteria	Fried frailty index. Domains: walk speed, grip strength, physical activity, exhaustion and weight	Engelhardt et al. (2017) [27] Holmes et al. (2014) [39] Muffly et al. (2013) [31] Muffly et al. (2014) [32] Ombres et al. (2022) [34] Pamukcuoglu et al. (2019) [35] Rodrigues et al. (2020) [40] Sung et al. (2024) [38]
GA	Geriatric assessment. Domains: physical function, comorbid medical conditions, cognition, nutritional status, psychological status, social activity, social support.	Pamukcuoglu et al. (2019) [35]
HCT‐CI	Hematopoietic cell transplantation comorbidity index. Domains: obesity, diabetes, cardiac impairments (arrhythmia, valve disease), cerebrovascular disease, psychiatric disturbance, renal, hepatic and pulmonary function, rheumatologic disease, peptic ulcer, infections requiring antibiotics, history of solid tumors.	Aydin et al. (2023) [24]
IMWG frailty score/IMWG frailty index^a^	International myeloma working group frailty score. International myeloma working group frailty index. Domains: age, comorbidity, activity of daily living and instrumental activities of daily living.	Belotti et al. (2020) [25] Holler et al. (2023) [28] Nathwani et al. (2020) [33]
mGA	Modified cancer specific geriatric assessment. Domains: comorbidity, patient‐reported functional status, performance or provider‐based functional status, social support, cognition, psychological, nutrition status, polypharmacy, biomarkers. Modifications in the current study: supplemented with 4 m walk and grip strength, time up and go, and short‐form 36.	Derman et al. (2019) [26]
MoCA	Montreal cognitive assessment battery. Domains: cognitive screening measure (sensitive to early impairments in memory and executive function), executive function (significant associated with frailty).	Smith et al. (2022) [37]
R‐MCI	Revised myeloma comorbidity index. Domains: renal and lung function, Karnofsky performance status, frailty and age, allows to include cytogenetics.	Engelhardt et al. (2017) [27] Holler et al. (2023) [28]
SPPB	Short physical performance battery. Domains: balance, timed 4 m walk and chair stands.	Smith et al. (2022) [37]
VES‐13	Vulnerable elders survey. Domains: age, self‐rated health and functional status (13 item survey).	Holmes et al. (2014) [39]

^a^ IMWG frailty score/IMWG frailty index is both used to describe the domains.

### 3.2 Frailty Score and Age

Six articles showed an association between higher age and the increased occurrence of impairments and comorbidities [25, 27, 28, 30, 31, 32, 34]. Sung et al. observed that the older age cohort showed a higher number of prefrail and frail patients compared with the younger cohort. Another article showed the presence of frailty among younger patients and that frailty increased with age. These results were confirmed in multivariable analysis [27]. Two articles found an association between older age and cognitive decline [30, 37]. Lew et al. also reported that patients aged 60 years and older had a higher risk for nutritional impairment compared with younger patients, and that nutritional impairment and older age could lead to impaired overall survival. Two articles showed no association between older age, impairments, and comorbidities [35, 36]. Moreover, three articles showed no association between patients considered eligible for HSCT and patients classified as fit or between patients considered ineligible for HSCT and patients classified as frail [25, 33, 37]. Two articles show that frailty and geriatric vulnerabilities may be present in patients already considered eligible for HSCT [38, 40].

### 3.3 The Extent of Frailty and Clinical Outcomes

The extent of frailty was categorized within the three subgroups of fit, prefrail/unfit/intermediate‐fit, and frail across the included articles. Thirteen articles presented their results of frailty based upon these subgroups, and frailty ranged from 7% to 38% [25, 27, 28, 29, 31, 32, 33, 34, 35, 36, 37, 38, 40]. Three articles reported the extent of frailty for two of the three subgroups: fit and frail/unfit/intermediate‐fit [24] and fit and frail [34, 35]. Patients classified as frail or frail/unfit/intermediate‐fit ranged from 21% to 46% [24, 34, 35]. Eleven articles described their patients as prefrail/unfit/intermediate‐fit, ranging from 25% to 60% [24, 25, 27, 28, 29, 31, 32, 33, 37, 38, 40]. Three articles did not report on the extent of frailty [26, 30, 39]. The prevalence of frailty is described in Table 5.

**TABLE 5:** Prevalence of frailty.

Author, (year), reference number	Frailty tool	*n* (%)		
		Fit	Prefrail/unfit/intermediate‐fit	Frail
Aydin et al. (2023) [24]	Geriatric‐8 screening tool Hematopoietic cell transplantation‐specific comorbidity index Acute myelocytic leukemia score	65 (54%)	55 (46%)	
Belotti et al. (2020) [25]	International myeloma working group frailty score	43 (33%)	79 (60%)	9 (7%)
Derman et al. (2019) [26]	Modified cancer specific geriatric assessment	Na	Na	Na
Engelhardt et al. (2017) [27]	Revised myeloma comorbidity index Fried frailty score	247 (31%)	446 (56%)	108 (14%)
Holler et al. (2023) [28]	Revised myeloma comorbidity index International myeloma working group frailty score	73 (29%)	145 (58%)	32 (13%)
Holmes et al. (2014) [39]	Vulnerable elders survey‐13 Fried criteria Geriatric‐8 screening tool	Na	Na	Na
Huang et al. (2023) [29]	Comprehensive geriatric assessment Deficit accumulation frailty index Blessed orientation‐memory‐concentration test	95 (64%)	37 (25%)	16 (11%)
Lew et al. (2022) [30]	Clinical pre‐transplantation optimization program	Na	Na	Na
Muffly et al. (2013) [31]	Fried frailty index	26 (24%)	57 (51%)	28 (25%)
Muffly et al. (2014) [32]	Fried frailty index	29 (19%)	87 (56%)	38 (25%)
Nathwani et al. (2020) [33]	International myeloma working group frailty score	64 (39%)	55 (33%)	46 (28%)
Ombres et al. (2022) [34]	Fried frailty index	39 (78%)		11 (22%)
Pamukcuoglu et al. (2019) [35]	Frailty score Geriatric assessment	77 (79%)		21 (21%)
Rodrigues et al. (2020) [40]	Fried phenotype criteria	14 (38%)	16 (43%)	7 (19%)
Salas et al. (2021) [36]	Clinical frailty score	103 (62%)		62 (38%)
Smith et al. (2022) [37]	Short physical performance battery Montreal cognitive assessment battery	50 (48%)	35 (34%)	19 (18%)
Sung et al. (2024) [38]	Fried frailty index	98 (35%)	161 (58%)	21 (8%)

Abbreviation: Na, not applicable.

Most articles (*n* = 9) aimed to assess whether frailty could predict morbidity and mortality. Six of these articles showed a significant association between fit patients and longer overall survival [24, 27, 28, 32, 38]. These results remained significant for AML‐score and G8‐score (not HCT‐CI score) in multivariable analysis in one study [24]. A study showed worse overall survival in patients with one or more impairments [30]. Another study found that non‐relapse mortality was the most common cause of death in relation to frailty, which significantly led to a decrease in deaths due to relapse of the underlying disease [38]. Two articles showed an association between frail patients and an increased risk of infection and serious adverse events of cardiac, pulmonary, and renal impairments [28, 35]. Engelhart et al. found that frailty, impaired renal function, and lung function were significant risk factors of overall survival in multivariable analysis [27]. Holler et al. showed an association between frailty and hematological complications, such as anemia and thrombocytopenia. However, they found no association between frailty and leukocytopenia. Two articles showed a significant association between patients being fit and progression‐free survival [25, 28]. These results were confirmed in multivariable analysis [25]. Three articles presented an association between patients being prefrail/unfit/intermediate‐fit and outcome [28, 29, 38]. Furthermore, two articles showed that patients classified as intermediate fit had better survival and progression‐free survival compared with frail patients [28, 29]. One article showed significantly worse overall survival for patients classified as prefrail compared to patients classified as fit, and higher rates of pneumonia, acute GvHD, and sepsis among prefrail patients compared to fit patients [38].

## 4 Discussion

This systematic review aimed to analyze the extent of frailty in patients with hematologic malignancies and those undergoing HSCT, and to explore the associations between frailty and age, and clinical outcomes. A variety of tools were used to predict frailty in these patients, each containing multiple domains, and these domains were mainly similar across the different tools. Inconsistency in how the outcomes of these domains were assessed makes it challenging to compare the various tools and to determine the number of frail patients as well as the association between frailty, morbidity, and mortality.

All patients in the included articles had various hematologic malignancies, and most patients were eligible for HSCT. In contrast, other reviews have included cancer patients in general, with a minority having hematological malignancies, and chemotherapy was the most common treatment [11, 13, 14]. While most of the tools in our review aimed to identify frailty, some were also designed to identify impairments or vulnerabilities, which often included aspects of frailty. Tools such as the Fried Frailty Index, G8 score, IMWG frailty score, and R‐MCI were frequently used in the articles in this review, while previous reviews included articles concerning GA [11, 13]. Domains such as nutritional status, comorbidities, social support, cognitive functioning, and physical activity were frequently included in our review. These domains are also included in GA; thus, systematic reviews limited to GA may per se not inherently diminish the understanding of frailty in recipients of HSCT. However, the operationalization of specific domains (i.e., nutritional status) may differ between the tools and may impact the results. For instance, the evaluation of nutritional status can be based on BMI, weight loss, physical signs of malnutrition (e.g., muscle wasting, edema), or biochemical markers of nutritional status. These variations may not be directly comparable and may impact the frailty score. We found that the categorization of frailty (i.e., frail or frail/unfit/intermediate‐fit) differed between the articles and may impact the proportion of frailty. In line with this, the proportion of patients categorized as frail ranged from 7% to 38%, while 21% to 46% were categorized as frail or frail/unfit/intermediate‐fit.

Our review indicates that the role of age in predicting frailty is conflicting. Some of the included articles revealed that frailty, vulnerability, and impairments increased with age, and that HSCT is particularly challenging among older patients [38]. In contrast, an included article found that frailty at baseline did not differ between the younger and older cohorts of HSCT‐eligible patients [35]. However, the patients included in the latter article were younger than the patients included in another article [38].

Our findings suggest an association between the tools used to identify frailty and outcomes, such as survival and toxicities. This aligns with a systematic review stating that the use of GA can identify vulnerabilities in older adults planned for HSCT that are not captured by traditional measures [15]. Therefore, conducting a GA prior to HSCT may uncover vulnerabilities, and these results could tailor pretransplantation interventions, potentially improving patient outcomes [15]. In line with other reviews [11, 13], we found an association between frailty and adverse treatment events. Frail patients exhibited an increased risk of infection and serious adverse events, including cardiac, pulmonary, and renal impairments. Moreover, the relationship between frailty and hematological toxicity (i.e., a decrease in bone marrow and blood cells may lead to infection, bleeding, or anemia) was explored. We found that anemia and thrombocytopenia were observed in frail patients, whereas no association between frailty and leukocytopenia was found [28]. Symptoms of anemia, such as feeling weak and tired, are captured by frailty assessment, whereas leukocytopenia without infectious complications may be non‐symptomatic. Similarly, a systematic review found that impairments in patients with hematologic malignancies may be associated with toxicity, thereby predicting clinical outcomes [11]. Additionally, we found that frailty was associated with non‐hematologic toxicity grades 3–4 (i.e., adverse events of the treatment affecting organs and systems outside the blood and bone marrow) measured 1 year after HSCT [35]. In contrast, one article reported the same rates of hematological and non‐hematological toxicity in patients considered fit, intermediate fit, or frail [33]. These conflicting results may be due to heterogeneous diagnoses, treatment modalities, age, assessment tools, and follow‐up times, which impact the assessment of toxicity. Furthermore, we found that frailty was associated with reduced overall survival, which aligns with three systematic reviews describing an association between impairments and a decline in overall survival [11, 15, 16].

A methodological strength of this review was that the literature search was conducted in highly relevant databases and that a hand search was performed. The search strategy was established with advice from an experienced research librarian utilizing search terms based on keywords employed in previous articles. Consequently, we captured a wide number of articles. Another strength was that the first authors independently assessed eligibility, appraised the methodological quality, and extracted data. Another strength was the inclusion of multiple tools to assess frailty, providing a broader understanding of frailty. This systematic review was also strengthened by limiting it to hematological malignancies and not cancer patients in general. Thus, the patient population was more homogeneous in relation to diagnosis and treatment modalities impacting frailty.

This systematic review has some limitations. Articles were included regardless of their quality. Several studies had a small sample size, and there was heterogeneity within the populations due to the variation in diagnosis, conditioning regimes for HSCT (i.e., toxicity of the chemotherapy), type of transplantation (i.e., auto‐HSCT and allo‐HSCT), age, frailty screening tools, and study outcome. The latter reflects methodological challenges within studies of adults with hematological malignancies undergoing HSCT. Moreover, multivariable analysis of interactions between covariates potentially affecting the results was limited by the small sample size. Baseline characteristics (e.g., disease stage, time from diagnosis to transplant) that may predict outcomes in frail patients were also limited. The studies used one or several tools to identify frailty, vulnerability, or impairments. However, several studies did not report on which specific clinical team member (i.e., physician, nurse, social worker) used the tool, or what kind of competence was needed for performing the screening.

This systematic review aimed to analyze the extent of frailty in patients with hematologic malignancies and patients undergoing HSCT, and explore the associations between frailty and age, and clinical outcomes. Seventeen tools were identified that were used to assess frailty, vulnerability, or impairment, and some of them were used in several studies. Frailty in recipients of HSCT was characterized by abnormal nutrition status, comorbidities, impact on social support, activities of daily living, low physical activity, low grip strength, low gait speed, weight loss, exhaustion, and cognitive decline. Further, there seemed to be an association between fit patients and longer overall and progression‐free survival and an association between frailty and morbidity and mortality in patients with hematologic malignancies and recipients of HSCT. HSCT among the older population is challenging, and studies suggest a clear connection between frailty and age.

Inclusion of frailty assessment in the traditional pretransplant risk assessment may improve the predictive value of pretransplant risk assessment and identify patients at risk of morbidity and mortality. Thus, identifying patients at risk of frailty pre‐HSCT may identify a window for tailored interventions and reduce HSCT‐associated morbidity and mortality. The use of multiple tools is recommended for frailty assessment in hematological malignancies. Validated disease‐specific tools such as the IMWG frailty score and R‐MCI in patients with myeloma, and the FFI in patients with myelodysplastic syndrome, acute leukemia, and lymphoma should be preferred for use in clinical practice. However, GA tools such as the VES‐13, G8‐score, and CFS combined with a comorbidity index may identify frail patients undergoing HSCT regardless of the underlying disease.

## Author Contributions

**Marit Bakken:** conceptualization, investigation, writing – original draft, methodology, validation, visualization, writing – review and editing, software, formal analysis, project administration, data curation. **Marie Roko Kallager:** conceptualization, investigation, writing – original draft, methodology, validation, visualization, writing – review and editing, software, formal analysis, project administration, data curation. **Marie Hamilton Larsen:** metodology, validation, visualization, writing – review and editing, data curation. **Simen A. Steindal:** methodology, validation, visualization, writing – review and editing, data curation. **Kristin J. Skaarud:** conceptualization, investigation, writing – original draft, methodology, visualization, writing – review and editing, formal analysis, project administration, data curation, supervision, validation.

## Funding

This work was supported by the Oslo University Hospital.

## Consent

There is no patient or public contribution, as this is a systematic review.

## Conflicts of Interest

The authors declare no conflicts of interest.

## Supporting information


**Data S1:** Supporting Information.

## Data Availability

Data sharing not applicable to this article as no datasets were generated or analysed during the current study.
