# Supplementary material for: Frailty in Patients With Hematologic Malignancies and Patients Undergoing Hematopoietic Stem Cell Transplantation: A Systematic Review
Source: Cancer Rep (Hoboken). 2026 Jan 9;9(1):e70456. doi: 10.1002/cnr2.70456 (PMC12789654; doi:10.1002/cnr2.70456)
Supplement: Supplementary file 1 — Data S1: Supporting Information. [file CNR2-9-e70456-s001.docx]

| **Synthesis Without Meta-analysis (SWiM)** | | | |
| --- | --- | --- | --- |
| **SWiM reporting item** | **Item description** | **Page in manuscript where item is reported** | **Other*** |
| Methods | | | |
| 1. Grouping studies for synthesis | 1a) Provide a description of, and rationale for, the groups used in the synthesis (e.g., groupings of populations, interventions, outcomes, study design) | 8-9 |  |
|  | 1b) Detail and provide rationale for any changes made subsequent to the protocol in the groups used in the synthesis | Na |  |
| 2. Describe the standardized metric and transformation methods used | Describe the standardized metric for each outcome. Explain why the metric(s) was chosen, and describe any methods used to transform the intervention effects, as reported in the study, to the standardized metric, citing any methodological guidance consulted | Na |  |
| 3. Describe the synthesis methods | Describe and justify the methods used to synthesize the effects for each outcome when it was not possible to undertake a meta-analysis of effect estimates | 8-9 |  |
| 4. Criteria used to prioritize results for summary and synthesis | Where applicable, provide the criteria used, with supporting justification, to select the specific studies, or a particular study, for the main synthesis or to draw conclusions from the synthesis (e.g., based on study design, risk of bias assessments, directness in relation to the review question) | 6-7 |  |
| 5. Investigation of heterogeneity in reported effects | State the method(s) used to examine heterogeneity in reported effects when it was not possible to undertake a meta-analysis of effect estimates and its extensions to investigate heterogeneity | 8-9 |  |
| 6. Certainty of evidence | Describe the methods used to assess certainty of the synthesis findings | 8-9 |  |
| 7. Data presentation methods | Describe the graphical and tabular methods used to present the effects (e.g., tables, forest plots, harvest plots). Specify key study characteristics (e.g., study design, risk of bias) used to order the studies, in the text and any tables or graphs, clearly referencing the studies included | 8-9 |  |
| Results | | | |
| 8. Reporting results | For each comparison and outcome, provide a description of the synthesized findings, and the certainty of the findings. Describe the result in language that is consistent with the question the synthesis addresses, and indicate which studies contribute to the synthesis | 8-32 |  |
| Discussion |  |  |  |
| 9. Limitations of the synthesis | Report the limitations of the synthesis methods used and/or the groupings used in the synthesis, and how these affect the conclusions that can be drawn in relation to the original review question | 33 |  |
| Abbreviations: N, not applicable; PRISMA, Preferred Reporting Items for Systematic Reviews and Meta-Analyses. *If the information is not provided in the systematic review, give details of where this information is available (e.g. protocol, other published papers (provide citation details), or website (provide the URL). | | | |

| **Search strategy (conducted 31.01.2024)** | |
| --- | --- |
| **Database** | **Search strategy** |
| **Cinahl** | S1: «stem cell transplantation» (= 14 383 results)  S2: (MH «Hematopoietic Stem Cell Transplantation») OR «hematopoietic stem cell transplantation» (=11 676 results)  S3: (MH «Bone Marrow Transplantation») OR «bone marrow transplantation» (= 5687 results)  S4: «hematological malignancies» (= 1578 results)  S5: «Hematologic malignancies» (=2124 results)  S6: «Hematologic malignancy» (=742 results)  S7: «Hematologic malignancies» (=1 results)  S8: (MH «Hematologic Neoplasms+) OR «Hematologic neoplasms» (=4479 results)  S9: S1 OR S2 OR S3 OR S4 OR S5 OR S6 OR S7 OR S8 (=25 128 results)  S10: (MH «Geriatrics+) OR «Geriatrics» (= 11 826 results)  S11: (MH «Geriatric Assessment+») OR «Geriatric Assessment» (=19 657 results)  S12: «AML-score» (= 3 results)  S13: «G8-score» (= 2 results)  S14: «HEMA-4» (= 0 results)  S15: «Frailty screening tool» (= 49 results)  S16: «Frailty assessment» (= 490 results)  S17: «Frailty» (= 13 828 results)  S18: (MH «Frailty Syndrome») OR «Frailty syndrome» (= 5131 results)  S19: «Frailty index» (= 1447 results)  S20: «Frailty older» (= 23 results)  S21: «Frailty elderly» (= 14 results)  S22: (MH «Frail Elderly») OR «Frail elderly» (= 9723 results)  S23: «Frailty scale» (= 615 results)  S24: «Clinical frailty scale» (= 534 results)  S25: «Vulnerable» (= 44 564 results)  S26: S10 OR S11 OR S12 OR S13 OR S14 OR S15 OR S16 OR S17 OR S18 OR S19 OR S20 OR S21 OR S22 OR S23 OR S24 OR S25 (= 90 452 results)  S27: S9 AND S26 (= 212 results) |
| **Embase** | S1: «Stem cell transplantation.mp. OR exp stem cell transplantation/» (=207261 results)  S2: «Hematopoietic stem cell transplantation.mp. OR exp hematopoietic stem cell transplantation/» (=96582 results)  S3: «Bone marrow transplantation.mp. OR exp bone marrow transplantation/» (=78448 results)  S4: «Hematological malignancies.mp.» (=21921 results)  S5: «Hematologic malignancies.mp.» (=22660 results)  S6: «Hematologic malignancy.mp. OR exp. Hematologic malignancy/» (=757442 results)  S7: «Hematologic malignancies.mp.» (=92 results)  S8: «Hematologic neoplasms.mp.» (=1665 results)  S9: 1 OR 2 OR 3 OR 4 OR 5 OR 6 OR 7 OR 8 (=935452 results)  S10: «Geriatrics.mp. OR exp geriatrics/» (=56029 results)  S11: «Geriatric Assessment.mp. OR exp geriatric assessment/» (=24355 results)  S12: «AML-score.mp.» (=16 results)  S13: «G8-score.mp.» (=288 results)  S14: «HEMA-4.mp.» (=9 results)  S15: «Frailty screening tool.mp.» (=159 results)  S16: «Frailty assessment.mp.» (=1821 results)  S17: «exp frailty/ OR Frailty.mp.» (=46455 results)  S18: «Frailty syndrome.mp.» (=1165 results)  S19: «frailty index.mp.» (=5563 results)  S20: «Frailty older.mp.» (=52 results)  S21: «Frailty elderly.mp.» (=45 results)  S22: «Frail elderly.mp. OR exp frail elderly/» (=15475 results)  S23: «Frailty scale.mp.» (=3030 results)  S24: «Clinical frailty scale.mp. OR exp Clinical Frailty Scale/» (=2672 results)  S25: «Vulnerable.mp.» (=172067 results)  S26: 10 OR 11 OR 12 OR 13 OR 14 OR 15 OR 16 OR 17 OR 18 OR 19 OR 20 OR 21 OR 22 OR 23 OR 24 OR 25 (=292115 results)  S27: 9 AND 26 (=5589 results) |
| **Medline** | S1: «Stem cell transplantation.mp. OR exp stem cell transplantation/» (=118038 results)  S2: «Hematopoietic stem cell transplantation.mp. OR exp Hematopoietic Stem Cell Transplantation/» (=66871 results)  S3: «Bone marrow transplantation.mp. OR exp Bone Marrow Transplantation/» (=54684 results)  S4: «Hematological malignancies.mp.» (=12108 results)  S5: «Hematologic malignancies.mp.» (=12363 results)  S6: «Hematologic malignancy.mp.» (=3951 results)  S7: «Hematologic malignancies.mp.» (=64 results)  S8: «Hematologic neoplasms.mp. OR exp Hematologic Neoplasms» (=26318 results)  S9: 1 OR 2 OR 3 OR 4 OR 5 OR 6 OR 7 OR 8 (=200158 results)  S10: «Geriatrics.mp. OR exp Geriatrics/» (=39315 results)  S11: «Geriatric Assessment.mp. OR exp Geriatric Assessment/» (=35105 results)  S12: «AML-score.mp.» (=6 results)  S13: «G8-score.mp.» (=110 results)  S14: «HEMA-4.mp.» (=7 results)  S15: «Frailty screening tool.mp.» (=87 results)  S16: «Frailty assessment.mp.» (=1069 results)  S17: «Frailty.mp. OR Frailty/» (=27907 results)  S18: «Frailty syndrome.mp.» (=774 results)  S19: «Frailty index.mp.» (=3255 results)  S20: «Frailty older.mp.» (=38 results)  S21: «Frailty elderly.mp.» (=21 results)  S22: «Frail elderly.mp. OR exp Frail Elderly/» (=17595 results)  S23: «Frailty scale.mp.» (=1291 results)  S24: «Clinical frailty scale.mp.» (=1101 results)  S25: «Vulnerable.mp.» (=133355 results)  S26: 10 OR 11 OR 12 OR 13 OR 14 OR 15 OR 16 OR 17 OR 18 OR 19 OR 20 OR 21 OR 22 OR 23 OR 24 OR 25 (=231603 results)  S27: 9 AND 26 (=963 results) |

| Quality assessment of the included articles | | | | | | | | | | | |
| --- | --- | --- | --- | --- | --- | --- | --- | --- | --- | --- | --- |
| JBI Quality assessment of the included articles with cohort studies. | | | | | | | | | | | |
| Author (year) | Q1 | Q2 | Q3 | Q4 | Q5 | Q6 | Q7 | Q8 | Q9 | Q10 | Q11 |
| Aydin et al. (2023) | Na | Na | Y | Na | Na | Y | Y | Y | U | Na | Y |
| Belotti et al. (2020) | Y | Y | U | N | N | Y | Y | U | Y | Na | Y |
| Derman et al. (2019) | N | Y | Y | Y | Y | Y | U | Y | U | N | Y |
| Engelhardt et al. (2017) | Y | Y | Y | Y | U | Y | Y | Y | Y | U | Y |
| Holler et al. (2023) | N | Y | Y | Y | Y | Y | Y | Y | Y | Na | Y |
| Huang et al. (2023) | Y | Y | Y | Y | N | Na | Y | Y | Y | Na | Y |
| Lew et al. (2022) | N | Y | Y | Y | N | Y | Y | U | U | N | Y |
| Muffly et al. (2013) | Y | Y | Y | Na | Na | Y | Y | Na | Na | Na | Y |
| Muffly et al. (2014) | Y | Y | Y | Y | Na | Y | Y | Y | Y | Na | Y |
| Nathwani et al. (2019) | Y | Y | U | Y | N | Y | U | Y | N | N | Y |
| Ombres et al. (2022) | Y | Y | Y | U | N | N | Y | Y | U | Na | Y |
| Pamukcuoglu et al. (2019) | N | Y | Y | N | N | N | Y | Y | Y | Na | Y |
| Salas et al. (2020) | U | Y | Y | N | N | Y | Y | Y | Y | N | Y |
| Smith et al. (2022) | Y | Y | Y | N | Na | Y | Y | U | Y | Na | Y |
| Sung et al.  (2024) | Y | Y | Y | Y | Y | Y | Y | Y | Y | Na | Y |
| Abbreviations: Questions, Q; Yes, Y; No, N; Unclear, N; Not applicable, Na.  Q1) Were the two groups similar and recruited from the same population?  Q2) Were the exposures measured similarly to assign people to both exposed and unexposed groups?  Q3) Was the exposure measured in a valid and reliable way?  Q4) Were confounding factors identified?  Q5) Were strategies to deal with confounding factors stated?  Q6) Were the groups/participants free of the outcome at the start of the study (or at the moment of exposure)?  Q7) Were the outcomes measured in a valid and reliable way?  Q8) Was the follow up time reported and sufficient to be long enough for outcomes to occur?  Q9) Was follow up complete, and if not, were the reasons to loss to follow up described and explored?  Q10) Were strategies to address incomplete follow up utilized?  Q11) Was appropriate statistical analysis used? | | | | | | | | | | | |
